# Analysis of participant-reported adverse events following the first dose of inactivated SARS-Cov-2 vaccine (TURKOVAC™) through telephone survey in Türkiye

**DOI:** 10.1080/07853890.2023.2183985

**Published:** 2023-03-13

**Authors:** Ateş Kara, Aslihan Coskun, Fehminaz Temel, Pervin Özelci, Selmur Topal, Ihsan Ateş

**Affiliations:** a Pediatric Infectious Disease Unit, Department of Pediatrics, Hacettepe University Faculty of Medicine, Ankara, Turkey; b Health Institutes of Türkiye, Turkish Vaccine Institute, Ankara, Turkey; c Field Epidemiology Unit, General Directorate of Public Health, Department of Communicable Diseases and Early Warning, Ministry of Health, Ankara, Turkey; d Ankara City Hospital, Internal Medicine Clinic, Ankara, Turkey

**Keywords:** COVID-19, vaccine, adverse events

## Abstract

**Background/Objective(s)/Introduction:**

TURKOVAC™ is a whole-virion inactivated COVID-19 vaccine, which was developed and recently granted emergency use authorization (conditional marketing authorization) in Türkiye. The objective of this study is to assess the spectrum and the distribution of adverse events reported following the administration of the first 150,000 doses as primary and booster vaccine doses in 22 state hospitals of 17 provinces in Türkiye.

**Patients/Materials and methods:**

In this cohort study, a verbal survey was conducted *via* telephone calls between 10 January and 17 January 2022, utilizing a structured questionnaire algorithm on a sample group of 20,000 persons on the third- and seventh-days following vaccination. The algorithm consisted of two parts focusing on both systemic and local adverse effects. Other adverse events reported by the participants were also recorded. 6023 people and 5345 people agreed to participate in the telephone survey on the 3rd- and 7th- days of having received the first dose of the vaccine, respectively.

**Results:**

Thirty-six-point-six percent of the participants on the 3rd day and 22.5% of the participants on the 7th day reported any adverse event following the first dose of the vaccine. On both follow-up days, the most commonly reported (29.7% for Day 3 and 13.1% for Day 7) adverse events were on the injection site. Among the local adverse events, the most frequently reported one was the pain on the injection site (27.9% for Day 3 and 12.4% for Day 7), induration (4.8% for Day 3 and 2.7% for Day 7) and swelling (3.5% for Day 3 and 2.0% for Day 7). Fatigue/weakness (9.6% for Day 3 and 8.3% for Day 7) and headache (7.9% for Day 3 and 8.0% for Day 7) were the most frequent systemic adverse events. Younger age, vaccine dose, and female sex were associated with having any adverse event and pain (on the injection site). Female sex was associated with more swelling (on the injection site), induration (on the injection site), fever, and a higher impact on daily living.

**Conclusion(s):**

In this study, we conducted a rapid assessment of adverse events following the first dose of the TURKOVAC vaccine. The vaccine appears to have a good safety profile in the first 7 days following vaccination. Younger age, vaccine dose, and female sex are associated with any adverse event and pain (on the injection site). These results present valuable information for the community and may contribute to increasing vaccine confidence.

**KEY MESSAGES**
As a whole-virion inactivated SARS-CoV-2 vaccine, the TURKOVAC™ vaccine, which has a favorable safety profile, can be an alternative to other COVID-19 vaccines including mRNA and viral vector vaccines.

## Introduction

Severe Acute Respiratory Syndrome Coronavirus 2 (SARS-CoV-2) was first detected in Wuhan, China in late December 2019. The outbreak was classified as a Public Health Emergency of International Concern (PHEIC) on 30 January 2021, and the World Health Organization (WHO) declared it a pandemic on 11 March 2021 [1].

One of the most essential strategies to control a pandemic is the rapid development of safe and effective vaccines. Isolation of the virus in 2019 accelerated the development of various vaccines by 2020. Several COVID-19 vaccines have been validated for use by the WHO and obtained emergency use approval as of 12 January 2022 [2]. As of 17 March 2022, a total of 10,925,055,390 COVID-19 vaccine doses have been administered worldwide [3].

During the pandemic, both inactivated CoronaVac and BNT162B2 mRNA vaccines have been used in primary vaccination in Turkey. Inactivated CoronaVac and BNT162B2 mRNA vaccinations were started on 13th January 2021, and 2nd April 2021 respectively. For individuals who have completed their primary vaccination with inactivated vaccines, the application of a booster dose is recommended at Month 6 as of July 2021.

Türkiye started its own COVID-19 vaccine development program in 2020. ERUCOV-VAC (later named TURKOVAC™), the preclinical study of which demonstrated high immunogenicity in BALB/c mice, complete protective effect against a lethal SARS-CoV-2 challenge in transgenic mice (K18-hACE2), and positively appreciable safety evaluation in the ferret models [4], is a whole-virion inactivated COVID-19 vaccine candidate with aluminum hydroxide adjuvant, produced by SBT Science and Biotechnologies. The dosage is 3 µg/0.5 ml per injection, and it is supplied in vials. TURKOVAC was granted emergency use authorization (conditional marketing authorization) on 22nd December 2021 in Türkiye. Phase III and IV trials are ongoing.

The first and most important step in ensuring the safety of vaccines is the continuous and voluntary reporting of adverse events following immunization (AEFI) [5]. Since there are various immunization strategies used in urban and rural settings and for diverse populations, adoption of the AEFI detection, investigation, and response strategies becomes crucial [6]. Therefore, the importance of quick AEFI assessment for community acceptance of recently introduced vaccines, including COVID-19 vaccines, in addition to the routine national AEFI monitoring system, is rather significant.

The objective of this study is to assess the spectrum and the distribution of adverse events experienced after the first dose of inactivated SARS-CoV-2 vaccine (TURKOVAC™) following the administration of the first 150,000 doses in 22 state hospitals of 17 provinces in Türkiye.

## Patients/materials and methods

**Figure 1.**
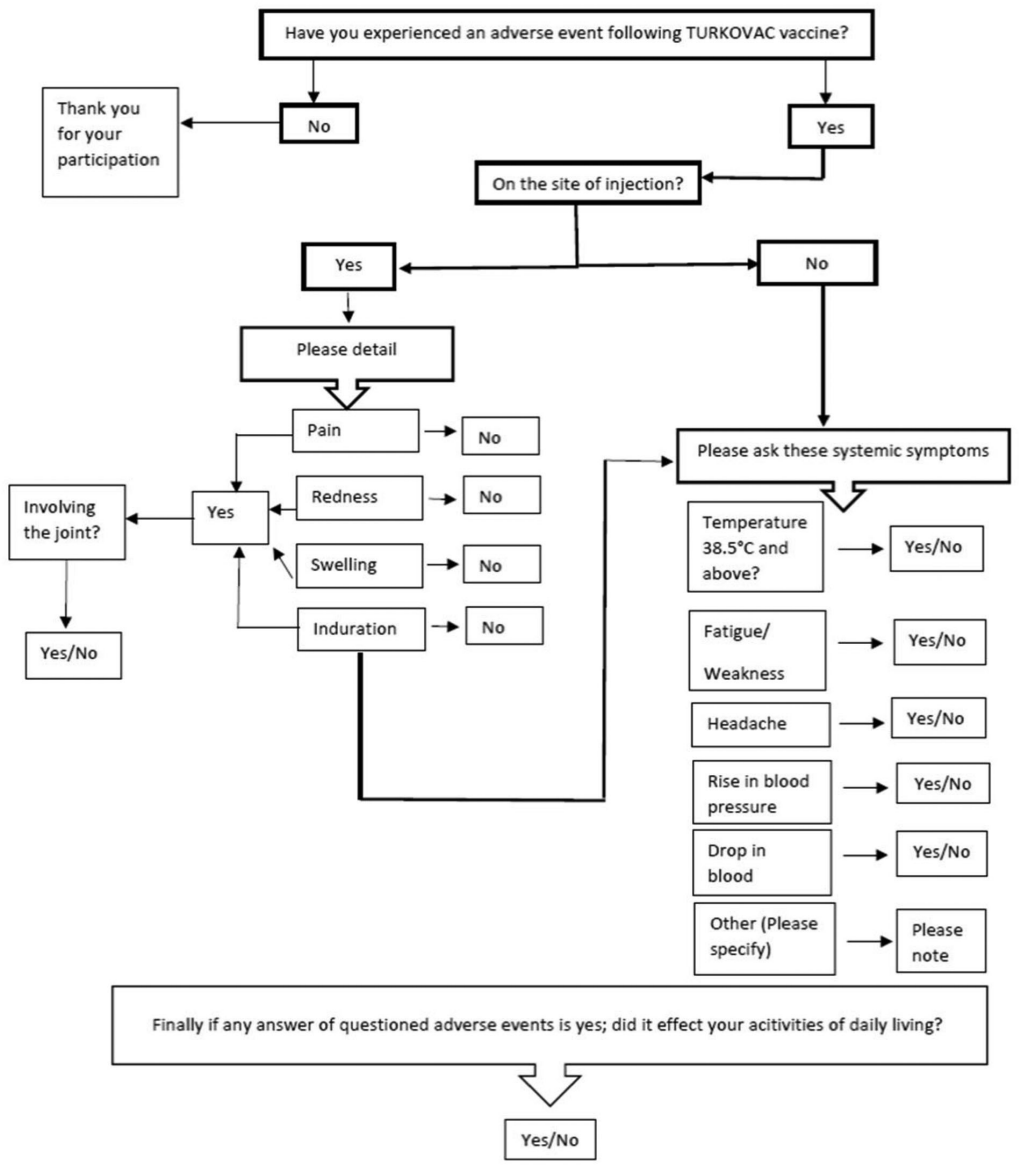
Questionnaire algorithm used by the call center during the telephone survey following the first dose TURKOVAC vaccine, Turkey, 10–17 January 2022. The ‘Other’ adverse events stated in the algorithm were open-ended. During the call, the ‘other’ adverse events reported by the participants were noted as open-ended text.

In this cohort study, a verbal survey was conducted *via* telephone calls between 10 January and 17 January 2022, utilizing a structured questionnaire algorithm on a sample group of 20,000 persons on the third and seventh days following vaccination (Figure 1).

The inclusion criteria included having been administered the TURKOVAC vaccine as the first dose of primary vaccination or as the booster dose following two-dose inactivated (CoronaVac) or two-dose mRNA (BNT162B2) vaccines as the primary vaccination protocol, and not having COVID-19 (confirmed by PCR) according to the official national electronic health recording system (HES). Prior COVID-19 vaccine doses and vaccine types were not asked.

The Ethics Committee approval permitted contacting the vaccinated individuals by phone as well as the administration of a questionnaire for safety monitoring. We had no access to any other data regarding previous vaccination or detailed medical history. Verbal consent was also obtained before the survey, at the beginning of the phone conversation.

People were contacted by Ministry of Health SABIM Call Centre personnel. The algorithm consisted of two parts focusing on both systemic and local adverse effects. Each person from the sample group was called (using the landline/mobile phone numbers they provided) three times, and if the calls were not answered, it was recorded as ‘no response’. 6023 people and 5345 people voluntarily agreed to participate in the telephone survey on the 3rd- and 7th- days of having received the first dose of the vaccine (either as primary vaccination dose or booster dose), respectively.

Participants were asked to answer if they experienced any adverse events following the first dose of the vaccine. If the answer was ‘yes’ the local and (pain, induration, swelling, and redness on the injection site) the systemic symptoms (fever, headache, fatigue/weakness, hypertension, hypotension) were probed in turn (Figure 1). These variables were questioned as they have been the most frequently reported adverse events in vaccine clinical trials including the phase trials of the TURKOVAC vaccine; and were among the most frequently reported adverse events associated with the inactivated and mRNA COVID-19 vaccines currently in use in Türkiye.

Local joint involvement was also probed to assess the severity of the local side effect. Furthermore, other adverse events reported by the participants were noted as open-ended text. Finally, each participant was asked if the adverse events they reported had influenced their daily life activities.

For sample size calculation, the proportion of any kind of adverse event or any kind of systemic or local events in the questionnaire was accepted as 50% (data was not available from previous studies) with a margin of error of 1% and a confidence interval (CI) of 95%. The sample size was calculated as 9500. 20,000 participants were intended to be surveyed to minimize the limitations of telephone surveys. 6023 people and 5345 people voluntarily agreed to participate in the telephone survey on the 3rd- and 7th- days of having received the first dose of the vaccine. The distribution of baseline characteristics of the participants by the day of follow-up was presented using frequency, percentage, mean ± SD, and median (min–max).

The adverse event rates were analyzed by sex, age groups, and vaccine dose, and relative risks (95% CI) were given for each adverse event and fisher, mid-p exact tests, and chi-square tests were used where necessary. The results are provided in the supplement. Any adverse event and all the adverse events were separately analyzed using two-sided statistical tests.

For selected adverse events, adverse event rates were presented separately for Day 3 and Day 7, and adjusted odds ratios (95% CI) were calculated by multivariate logistic regression analysis based on the age group, sex, and vaccine dose. Statistical significance was accepted as 5%.

The study was approved by Ankara City Hospital Ethics Committee No:2. All data were analyzed using SPSS software (version-23, IBM Corp., Armonk, N.Y., USA).

## Results

The baseline characteristics of the participants are presented in Table 1. 6023 people participated in the telephone survey on Day 3 of vaccination. Of these participants, 59.5% (3585) were male, and 40.5% (2438) were female. 83.2% were under the age of 65. The predominant age group is 40–55 years (35.3%) (mean ± SD: 50 ± 14.4, median (Min–Max): 51 (19–91)). 5345 people participated in the telephone survey on Day 7 of vaccination. Of these participants 60.5% (3233) were male, and 39.5% (2112) were female. 83.5% were under the age of 65. The predominant age group was 40–55 years (35.7%) (mean ± SD: 50.1 ± 14.2 median (min–max): 51 (19–89)) (Table 2).

**Table 1.** Baseline characteristics of the participants by follow-up day.

	Day 3 (*n* = 6023)		Day 7 (*n* = 5345)	
	(*n*)	(%)	(*n*)	(%)
Sex				
Male	3585	59.5	3233	60.5
Female	2438	40.5	2112	39.5
Age group				
19–39	1513	25.1	1310	24.5
40–55	2128	35.3	1909	35.7
56–64	1371	22.8	1243	23.3
65–75	878	14.6	768	14.4
76+	133	2.2	115	2.2
Mean ± SD	50 ± 14.4		50.1 ± 14.2	
Med (min–max)	51 (19–91)		51 (19–89)	
Vaccine dose^a^				
1	728	12.1	632	11.8
3	3444	57.2	3064	57.3
4	1700	28.2	1516	28.4
5	151	2.5	133	2.5

^a^The term ‘vaccine dose’ is used to define the number of doses of total COVID-19 vaccines including the TURKOVAC vaccine as the latest dose.

**Table 2.** Sex and age distribution of the participants by follow-up day.

	Day 3 (*n* = 6023)	Day 7 (*n* = 5345)
Male	50.7 ± 14.4	50.7 ± 14.3
	52 (19–91)	52 (19–89)
Female	49.0 ± 14.3	49.3 ± 14.0
	50 (19–86)	51 (19–86)
Total	50 ± 14.4	50.1 ± 14.2
	51 (19–91)	51 (19–89)

**Figure 2.**
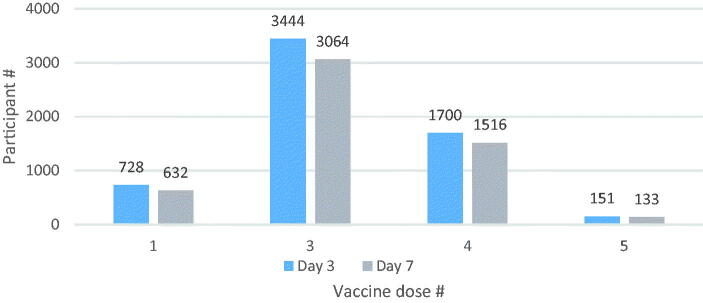
Distribution of participants by the number of doses of total COVID-19 vaccines (including the TURKOVAC vaccine as the latest dose) by follow-up days.

The TURKOVAC vaccine was administered as either the first dose of primary vaccination (12.1%) or as a booster dose (87.9%). No participant had received the TURKOVAC vaccine as the second dose (Figure 2).

The term ‘vaccine dose’ was used to define the number of doses of total COVID-19 vaccines including the TURKOVAC vaccine as the latest dose (e.g. ‘1’ stands for without any previous vaccination, and TURKOVAC vaccine was administered as the first dose of primary vaccination; ‘3’ stands for TURKOVAC booster following a two-dose primary vaccination with CoronaVac or BNT162B2).

Rates of adverse events reported by the participants by follow-up day are presented in Table 3. About 36.6% of the respondents on the 3rd day and 22.5% of the respondents on the 7th day reported any adverse events following the first dose of the vaccine. On both follow-up days, the most reported (29.7% for Day 3 and 13.1% for Day 7) adverse events were on the injection site. Among the local adverse events, the most frequent was pain on the injection site (27.9% for Day 3 and 12.4% for Day 7), induration (4.8% for Day 3 and 2.7% for Day 7) and swelling (3.5% for Day 3 and 2.0% for Day 7).

**Table 3.** Rates of adverse events reported by the participants by follow-up day.

Adverse event	Day 3 (*n* = 6023)		Day 7 (*n* = 5345)	
	*n*	Adverse event rate%	*n*	Adverse event rate%
Any adverse event	2207	36.6	1201	22.5
Local				
On the injection site	1789	29.7	698	13.1
Pain	1680	93.9	662	94.8
Induration	290	16.2	145	20.8
Swelling	213	11.9	105	15.0
Redness	49	2.7	42	6.0
Involvement of joint				
Pain	351	20.9	200	30.2
Induration	61	21.0	58	40.0
Swelling	36	16.9	34	32.4
Redness	7	14.3	14	33.3
Systemic				
Headache	479	7.9	426	8.0
Fatigue/Weakness	577	9.6	441	8.3
Other	285	4.7	121	2.3
Self-reported fever (≥38 °C)	67	1.1	76	1.4
Rise in blood pressure	63	1.0	74	1.4
Drop in blood pressure	45	0.7	57	1.1
Influence on daily living	247	4.1	233	4.4

Of the participants who reported having pain on the injection site, 20.9% (351/1680) reported that the pain involved the joint on Day 3 and 30.2% (220/662) reported that the pain involved the joint on Day 7.

Among the local adverse events, while the least frequent (2.7% for Day 3 and 6.0% for Day 7) was redness on the injection site, 14.3% also reported that the redness involved the joint on Day 3 and 33.3% reported that the pain involved the joint on Day 7.

Involvement of the joint was reported more frequently on Day 7. The most common systemic adverse events were fatigue/weakness (9.6% for Day 3 and 8.3% for Day 7) and headache (7.9% for Day 3 and 8.0% for Day 7). Fever was reported as an adverse event only by 1.1% of the respondents on Day 3 and by 1.4% on Day 7. Other systemic adverse events reported were quite similar on both follow-up days.

Only 4.1% of the participants on Day 3 and 4.4% on Day 7 reported that adverse events impacted their daily activities. Other adverse events were reported by only 4.7% and 2.3% of the respondents on Day 3 and Day 7 respectively. Other adverse events are given in detail in the supplement.

Comparative analysis of adverse events among participants who received TURKOVAC as their first COVID-19 vaccine compared to participants who received TURKOVAC as a booster dose is presented in Table 4. Age-, sex-, and vaccine dose-adjusted odds are presented in Table 5 for selected adverse events following the first dose of the TURKOVAC vaccine. Younger age, vaccine dose, and female sex were associated with any adverse event and pain (on the injection site). Female sex was associated with swelling (on the injection site), induration (on the injection site), fever, and impact on daily living. On both follow-up days, the rate of any adverse events reported was significantly higher (approx. 3 times) in the 19–39 age group compared to the 76+, 2 times higher among females compared to males, and 2 times higher at the first dose of the vaccine.

**Table 4.** Comparative analysis of adverse events among participants who received TURKOVAC as their first COVID-19 vaccine compared to participants who received TURKOVAC as a booster dose.

	Covariates	Day 3			Day 7		
		Adverse Event Rate %	RR (95% CI)	p	Adverse Event Rate %	RR (95% CI)	p
Any adverse event							
Vaccine dose	TURKOVAC as the 1st dose	48.2	**1.4 (1.3–1.5)**	**<0.001**	29.4	**1.4 (1.2–1.6)**	**<0.001**
	TURKOVAC as a booster dose^a^	35.1	1		21.5	1	
Pain							
Vaccine dose	TURKOVAC as the 1st dose	36.7	**1.4 (1.2–1.5)**	**<0.001**	16.1	**1.4 (1.1–1.7)**	**0.002**
	TURKOVAC as a booster dose^a^	26.7	1		11.9	**1**	
Redness							
Vaccine dose	TURKOVAC as the 1st dose	1.6	**2.4 (1.2–4.5)**	**0.008**	1.1	1.5 (0.7–3.3)	0.333
	TURKOVAC as a booster dose^a^	0.7	1		0.7	1	
Swelling							
Vaccine dose	TURKOVAC as the 1st dose	5.2	**1.6 (1.1–2.2)**	**0.009**	3.3	**1.9 (1.2–3.0)**	**0.009**
	TURKOVAC as a booster dose^a^	3.3	1		1.8	1	
Induration							
Vaccine dose	TURKOVAC as the 1st dose	6.7	**1.5 (1.1–2.0)**	**0.010**	4.0	**1.6 (1.1–2.4)**	**0.041**
	TURKOVAC as a booster dose^a^	4.6	1		2.5	1	
Fatigue/Weakness							
Vaccine dose	TURKOVAC as the 1st dose	13.7	1.5 (1.3–1.9)	**<0.001**	12.2	**1.6 (1.3–2.0)**	**<0.001**
	TURKOVAC as a booster dose^a^	9.0	1		7.7	1	
Headache							
Vaccine dose	TURKOVAC as the 1st dose	11.4	1.5 (1.2–1.9)	**<0.001**	11.7	**1.6 (1.2–2.0)**	**<0.001**
	TURKOVAC as a booster dose^a^	7.5	1		7.5	**1**	
Self-reported fever (≥38 °C)							
Vaccine dose	TURKOVAC as the 1st dose	1.6	1.6 (0.9–3.0)	0.141	2.2	1.7 (1.0–3.0)	0.073
	TURKOVAC as a booster dose^a^	1.0	1		1.3	1	
Drop in blood pressure							
Vaccine dose	TURKOVAC as the 1st dose	1.5	2.4 (1.2–4.6)	0.011	1.7	1.8 (0.9–3.4)	0.079
	TURKOVAC as a booster dose^a^	0.6	1		1.0	1	
Rise in blood pressure							
Vaccine dose	TURKOVAC as the 1st dose	1.1	1.1 (0.5–2.2)	0.881	0.8	0.5 (0.2–1.3)	0.174
	TURKOVAC as a booster dose^a^	1.0	1		1.5	1	
Impact on daily living							
Vaccine dose	TURKOVAC as the 1st dose	7.1	1.9 (1.4–2.6)	**<0.001**	7.6	**1.9 (1.4–2.6)**	**<0.001**
	TURKOVAC as a booster dose^a^	3.7	1		3.9	1	

^a^As primary vaccination or first or second booster with other vaccines, and TURKOVAC as the last booster dose.

Statistically significant one is given as bold.

**Table 5.** Adjusted odds for selected adverse events following the first dose of TURKOVAC vaccine.

	Covariates	Day 3		Day 7	
		Adverse event rate %	OR_ADJ_ (95% CI)	Adverse event rate %	OR_ADJ_ (95% CI)
Any adverse event					
Age	19–39	43.4	2.9 (1.9–4.5)	26.1	2.7 (1.5–4.9)
	40–55	38.7	2.5 (1.6–3.9)	23.8	2.5 (1.3–4.5)
	56–64	32.5	1.8 (1.2–2.8)	20.9	2.0 (1.1–3.6)
	65–75	28.8	1.5 (1.0–2.4)	17.1	1.5 (0.8–2.8)
	76+	20.3	1	11.3	1
Sex	Male	30.3	1	18.3	1
	Female	46.0	1.9 (1.7–2.1)	28.9	1.8 (1.6–2.0)
Vaccine dose	1	48.2	2.1 (1.4–3.2)	29.4	2.2 (1.2–3.9)
	3	36.2	1.4 (0.9–2.1)	22.0	1.6 (0.9–2.8)
	4	33.8	1.7 (1.2–2.6)	21.4	2.0 (1.2–3.5)
	5	21.9	1	11.3	1
Pain (on the injection site)					
Age	19–39	34.0	2.7 (1.7–4.4)	14.8	2.2 (1.1–4.5)
	40–55	29.1	2.2. (1.4–3.7)	13.2	2.0 (.0–4.0)
	56–64	24.1	1.6 (1.0–2.6)	11.3	1.5 (0.7–3.0)
	65–75	22.1	1.4 (0.9–2.3)	8.6	1.0 (0.5–2.1)
	76+	16.5	1	7.8	1
Sex	Male	22.1	1	9.7	1
	Female	36.4	2.0 (1.8–2.3)	16.5	1.8 (1.5–2.1)
Vaccine dose	1	36.7	2.0 (1.2–3.2)	16.1	3.1 (1.2–7.7)
	3	27.5	1.4 (0.9–2.2)	11.8	2.3 (0.9–5.8)
	4	25.9	1.7 (1.1–2.7)	12.7	3.5 (1.4–8.6)
	5	16.6	1	3.8	1
Swelling (on the injection site)					
Age	19–39	5.0	2.3 (0.7–7.9)	2.1	0.9 (0.2–3.3)
	40–55	3.2	1.5 (0.4–5.1)	2.3	1.1 (0.3–4.0)
	56–64	3.1	1.2 (0.4–4.2)	1.6	0.6 (1.2–2.0)
	65–75	2.7	1.1 (0.3–3.7)	1.4	0.4 (0.1–1.6)
	76+	2.3	1	2.6	1
Sex	Male	1.6	1	0.8	1
	Female	6.4	4.1 (3.0–5.7)	3.7	4.7 (3.0–7.3)
Vaccine dose	1	5.2	2.3 (0.5–10.1)	3.3	2.4 (0.3–18.9)
	3	3.2	1.7 (0.4–7.0)	1.5	1.0 (0.1–8.0)
	4	3.6	2.5 (0.6–10.5)	2.5	2.7 (0.4–20.2)
	5	1.3	1	0.8	1
Induration (on the injection site)					
Age	19–39	5.8	7.3 (1.0–53.9)	2.8	1.1 (0.3–3.9)
	40–55	5.5	7.3 (1.0–53.7)	3.1	1.3 (0.4–4.5)
	56–64	3.9	4.8 (0.7–35.8)	1.9	0.7 (0.2–2.3)
	65–75	3.5	4.6 (0.6–33.8)	2.7	0.9 (0.3–3.2)
	76+	0.8	1	2.6	1
Sex	Male	2.7	1	1.4	1
	Female	7.9	3.1 (2.4–4.0)	4.7	3.5 (2.5–5.1)
Vaccine dose	1	6.7	2.2 (0.7–7.5)	4.0	1.8 (0.4–8.0)
	3	4.8	1.7 (0.5–5.5)	2.3	1.1 (0.3–4.5)
	4	4.2	1.9 (0.6–6.2)	3.0	1.7 (0.4–7.2)
	5	2.0	1	1.5	1
Fatigue/Weakness					
Age	19–39	12.9	0.4 (0.2–0.9)	10.4	0.6 (0.3–1.4)
	40–55	9.0	0.6 (0.3–1.4)	8.2	0.7 (0.3–1.8)
	56–64	8.3	0.6 (0.3–1.5)	6.9	0.9 (0.4–2.1)
	65–75	8.1	0.6 (0.3–1.4)	7.4	0.8 (0.3–1.8)
	76+	4.5	1	5.2	1
Sex	Male	6.2	1	5.4	1
	Female	14.5	0.4 (0.3–0.5)	12.6	0.4 (0.3–0.5)
Vaccine dose	1	13.7	0.5 (0.2–1.1)	12.2	0.2 (0.1–0.8)
	3	9.6	0.7 (0.3–1.4)	7.9	0.3 (0.1–1.1)
	4	8.2	0.7 (0.3–1.5)	7.8	0.3 (0.1–1.0)
	5	5.3	1	2.3	1
Headache					
Age	19–39	9.3	0.5 (0.2–1.3)	8.9	0.2 (0.1–1.0)
	40–55	8.3	0.5 (0.2–1.4)	8.6	0.2 (0.1–1.0)
	56–64	7.1	0.5 (0.2–1.5)	7.2	0.3 (0.1–1.1)
	65–75	6.7	0.5 (0.2–1.3)	6.9	0.3 (0.1–1.1)
	76+	3.0	1	1.7	1
Sex	Male	5.0	1	5.5	1
	Female	12.3	0.4 (0.3–0.5)	11.8	0.5 (0.4–0.5)
Vaccine dose	1	11.4	0.3 (0.1–0.8)	11.7	0.3 (0.1–0.9)
	3	8.2	0.4 (0.1–1.0)	7.8	0.5 (0.2–1.3)
	4	6.4	0.5 (0.2–1.3)	7.3	0.5 (0.2–1.2)
	5	2.6	1	3.0	1
Self-reported fever (≥38 °C)					
Age	19–39	1.3	0.8 (0.3–2.0)	1.2	0.7 (0.3–1.7)
	40–55	1.0	0.7 (0.3–1.7)	1.8	1.1 (0.5–2.4)
	56–64	1.2	0.9 (0.4–2.2)	1.0	0.6 (0.3–1.4)
	65+	0.9	1	1.5	1
Sex	Male	0.5	1	0.8	1
	Female	2.0	3.8 (2.2–6.4)	2.4	3.2 (1.9–5.1)
Vaccine dose	1	1.6	1.1 (2.2–5.4)	2.2	2.3 (0.3–18.8)
	3	1.2	0.9 (0.2–3.7)	1.3	1.3 (0.2–10.3)
	4	0.7	0.4 (0.1–2.0)	1.4	1.5 (0.2–11.2)
	5	1.3	1	0.8	1
Impact on daily living					
Age	19–39	6.4	1.4 (0.5–4.0)	6.3	1.7 (0.6–4.8)
	40–55	4.5	1.0 (0.4–2.9)	4.3	1.2 (0.4–3.4)
	56–64	2.2	0.5 (0.2–1.6)	3.0	0.7 (0.3–2.1)
	65–75	2.4	0.7 (0.2–2.1)	3.6	0.9 (0.3–2.6)
	76+	3.0	1	3.5	1
Sex	Male	2.3	1	2.6	1
	Female	6.8	3.1 (2.4–4.1)	7.1	2.9 (2.2–3.7)
Vaccine dose	1	7.1	2.0 (0.6–6.6)	7.6	–
	3	4.4	1.4 (0.4–4.6)	3.9	–
	4	2.5	1.1 (0.3–3.5)	4.4	–
	5	2.0	1	0.0	1

On both follow-up days, pain (on the injection site) was significantly more common (approx. 3 times) in the 19–39 age group compared to the 76+, 2 times higher among females compared to males. The pain was twice more common on Day 3 and 3.1 times more common on Day 7 at the first dose of the vaccine. Swelling (on the injection site) rates were significantly higher (approx. 4 times) on both follow-up days among females compared to males. The rates of induration (on the injection site), fever, and impact on daily activities were significantly higher (approx. 3 times) on both follow-up days among females compared to males.

## Discussion

In this study, we conducted a rapid assessment of adverse events following the first dose of the TURKOVAC™ vaccine. In our study, only 16.8% of the participants were under 65 years of age although the elderly is the most vulnerable and among the top priority population for COVID-19. Inactivated CoronaVac and BNT162B2 mRNA vaccinations were started on 13 January 2021 and 2 April 2021, respectively. The elderly population was among the primary risk groups when COVID-19 vaccination was launched. Since this group had already been vaccinated with primary and even booster doses of inactivated and mRNA vaccines that were currently in use in Türkiye; the percentage of the participants above 65 years of age who were vaccinated with the TURKOVAC vaccine was relatively low.

Thirty-six-point-six percent of the participants on the 3rd day and 22.5% of the participants on the 7th day reported any adverse events following the first dose of the TURKOVAC vaccine. On both follow-up days, the most frequently reported (29.7% for Day 3 and 13.1% for Day 7) adverse event was on the injection site. Aluminum adjuvant-containing vaccines can cause injection site pain and tenderness, post-immunization headache, arthralgia, and myalgia [7]. The widely used dose of aluminum hydroxide-based adjuvant is 0.5 mg/dose (based on aluminum ion content). The recommended amount of aluminum hydroxide-based adjuvant by WHO is ≤1.25 mg of aluminum ion per dose [8]. The amount of aluminum hydroxide in the TURKOVAC vaccine is 0.5 mg per dose.

Fever was reported as an adverse event only by 1.1% of the respondents on Day 3 and 1.4% of the respondents on Day 7, which was lower than expected. This finding is likely to be associated with self-reporting since taking temperature is not a recommended approach following vaccination.

In our study, the most frequent local adverse events were pain on the injection site (27.9% for Day 3 and 12.4% for Day 7), induration (4.8% for Day 3 and 2.7% for Day 7), and swelling (3.5% for Day 3 and 2.0% for Day 7). Fatigue/weakness (9.6% for Day 3 and 8.3% for Day 7) and headache (7.9% for Day 3 and 8.0% for Day 7)were the most frequent systemic adverse events reported by the participants. In the double-blind, randomized, placebo-controlled, phase 3 trial study of the CoronaVac vaccine in Turkey, the most common systemic adverse event was fatigue (546 [8.2%] participants in the vaccine group and 248 [7.0%] in the placebo group, *p* = 0.0228). Injection-site pain was the most frequent local adverse event (157 [2.4%] in the vaccine group and 40 [1.1%] in the placebo group, *p* < 0.0001) [9]. These findings are compatible with common side effects of COVID-19 vaccines including pain at the injection site, fever, fatigue, headache, muscle pain, chills, and diarrhea [10,11].

Other adverse events were reported by only 4.7% and 2.3% of the respondents on Day 3 and Day 7, respectively. The most frequently reported other adverse events on Day 3 were nausea, arthralgia, flu-like illness, chills and sore throat. On Day 7, sore throat, arthralgia, flu-like illness, chest pain, and myalgia were the most frequent other adverse events reported. No other adverse events requiring hospital admission were reported.

The factors associated with adverse effects were younger age, vaccine dose, and female sex (Table 5). In our study, younger age was significantly associated with any adverse event and pain (on the injection site). A progressive functional decline in the immune system occurs with age [12].

In our study, female sex was significantly associated with swelling (on the injection site), induration (on the injection site), fever, and impact on daily living. In a nationwide survey of the Comirnaty vaccine in Israel carried out between December 2021 and March 2022, participants who were younger than 40 and of female sex reported adverse events more frequently than others [13]. In a retrospective study of approximately 7000 reports submitted to the Centers for Disease Control and Prevention Vaccine Adverse Event Reporting System (recorded between December 2020 and January 2021), more than 79% of the reports were for female participants. The most frequently reported adverse events were headache, fatigue, and dizziness. This could be related to the higher frequency of CD4+ T cells among women. Hormones may also influence the immune responses of men and women since some immune cells have estrogen receptors [14]. Women also show a higher humoral and cell-mediated immune response to antigenic stimulation, vaccination, and infection compared to men. Both the baseline levels of immunoglobulin (Ig) and antibody responses to viruses and vaccines are consistently higher in females than in males among both younger and older populations [15]. In a study, women developed greater cytokine and antibody responses compared to men after getting the flu vaccine [16]. This result might also be related to reporting issues; women may be experiencing vaccine side effects more than men, but men may also be reporting them less frequently.

Training the interviewers on using a structured algorithm before the survey to standardize and minimize the bias is a strength of this study. A significant portion (88.7%) of the respondents on Day 3 (6023) could be reached on Day 7 (5345), which was also an advantage for the study. This study has some limitations. Only three factors (age, sex, vaccine dose) could be assessed; other factors such as vaccine type and adverse events of previous COVID-19 vaccination and underlying diseases could provide a more detailed perspective.

Telephone surveys have several advantages over face-to-face interviewing, such as providing a geographically widely distributed sample and inclusion of additional participants living in remote areas, providing a rapid and high population coverage, and lower costs per interview [16].

Representativeness might be a limitation for telephone surveys [16]. In our study, the sample was selected from a group of people using either landline or mobile phone numbers they provided. This might be regarded as a weakness of the study. Using self-reporting as the main method might have led to bias regarding the validity (severity) of the adverse events.

## Conclusion

Based on the telephone survey conducted to assess participant-reported short-term adverse effects following the first dose of the TURKOVAC™ vaccine, the vaccine seems to have a good safety profile in the first 7 days of vaccination. One-third of the participants reported any adverse events following the first dose. On both follow-up days, the most frequently reported adverse events were on the injection site, which was consistent with the phase trial results. Fatigue/weakness and headache were the most frequent systemic adverse events. Younger age, vaccine dose, and female sex were associated with having any adverse events and pain (on the injection site). These results present valuable information for the community and may contribute to increasing vaccine confidence. Larger studies may provide more detailed data.

## Data Availability

Raw data were generated at SABIM. Derived data supporting the findings of this study are available on request from the corresponding author, A.K. The data are not publicly available as the information contained may compromise the privacy of research participants.

## References

[1] Timeline: WHO's COVID-19 response. https://www.who.int/emergencies/diseases/novel-coronavirus-2019/interactive-timeline.

[2] Coronavirus disease (COVID-19): vaccines. https://www.who.int/news-room/questions-and-answers/item/coronavirus-disease-(covid-19)-vaccines.

[3] WHO coronavirus (COVID-19) dashboard. https://covid19.who.int.

[4] Pavel STI, Yetiskin H, Uygut MA, et al. Development of an inactivated vaccine against SARS CoV-2. Vaccines (Basel). 2021;9(11):1266. 34835197 10.3390/vaccines9111266 PMC8624180

[5] Adverse Events Following Immunization (AEFI). https://www.who.int/teams/regulation-prequalification/regulation-and-safety/pharmacovigilance/health-professionals-info/aefi.

[6] COVID-19 vaccines: safety surveillance manual. 2nd ed. Geneva: World Health Organization; 2021. Licence: CC BY-NC-SA 3.0 IGO.

[7] Petrovsky N. Comparative safety of vaccine adjuvants: a summary of current evidence and future needs. Drug Saf. 2015;38(11):1059–1074. 26446142 10.1007/s40264-015-0350-4 PMC4615573

[8] He P, Zou Y, Hu Z. Advances in aluminum hydroxide-based adjuvant research and its mechanism. Hum Vaccin Immunother. 2015;11(2):477–488. 25692535 10.1080/21645515.2014.1004026 PMC4514166

[9] Tanriover MD, Doğanay HL, Akova M, et al. Efficacy and safety of an inactivated whole-virion SARS-CoV-2 vaccine (CoronaVac): interim results of a double-blind, randomised, placebo-controlled, phase 3 trial in Turkey. Lancet. 2021;398(10296):213–222. Epub 2021 Jul 8. Erratum in: lancet. 2022 Jan 29;399(10323):436. 34246358 10.1016/S0140-6736(21)01429-X PMC8266301

[10] Side effects of COVID-19 vaccines. [updated 2021 Mar 31; cited 2022 Mar 21]. https://www.who.int/news-room/feature-stories/detail/side-effects-of-covid-19-vaccines.

[11] Possible Side Effects After Getting a COVID-19 vaccine. [updated 2022 Jan 12; cited 2022 Mar 21] https://www.cdc.gov/coronavirus/2019-ncov/vaccines/expect/after.html.

[12] Fink AL, Klein SL. Sex and gender ımpact ımmune responses to vaccines among the elderly. Physiology. 2015;30(6):408–416. 26525340 10.1152/physiol.00035.2015 PMC4630198

[13] Shapiro Ben DS, Gez Sharon B, Daniella R-C, et al. Immediate side effects of comirnaty COVID-19 vaccine: a nationwide survey of vaccinated people in Israel, december 2020 to march 2021. Euro Surveill. 2022;27(13):pii = 2100540. 10.2807/1560-7917.ES.2022.27.13.2100540 PMC8973016 35362408

[14] Women report more side effects from the COVID-19 vaccine than men. Health experts explain why. 2021. https://www.usatoday.com/story/news/health/2021/04/10/covid-vaccine-women-report-more-side-effects-than-men-heres-why/7139366002/.

[15] Potluri T, Fink AL, Sylvia KE, et al. Age-associated changes in the impact of sex steroids on influenza vaccine responses in males and females. NPJ Vaccines. 2019;4:29. Erratum in: NPJ Vaccines. 2019 Aug 12;4:35. 31312529 10.1038/s41541-019-0124-6 PMC6626024

[16] Boland M, Sweeney M, Scallan E, et al. Emerging advantages and drawbacks of telephone surveying in public health research in Ireland and the U.K. BMC Public Health. 2006;6(1):208. 16911771 10.1186/1471-2458-6-208 PMC1560130

